# Targeting of YAP1 by microRNA-15a and microRNA-16-1 exerts tumor suppressor function in gastric adenocarcinoma

**DOI:** 10.1186/s12943-015-0323-3

**Published:** 2015-02-22

**Authors:** Wei Kang, Joanna HM Tong, Raymond WM Lung, Yujuan Dong, Junhong Zhao, Qiaoyi Liang, Li Zhang, Yi Pan, Weiqin Yang, Jesse CS Pang, Alfred SL Cheng, Jun Yu, Ka Fai To

**Affiliations:** Department of Anatomical and Cellular Pathology, State Key Laboratory of Oncology in South China, Prince of Wales Hospital, The Chinese University of Hong Kong, Hong Kong, SAR PR China; Institute of Digestive Disease, Partner State Key Laboratory of Digestive Disease, The Chinese University of Hong Kong, Hong Kong, SAR PR China; Li Ka Shing Institute of Health Science, Sir Y.K. Pao Cancer Center, The Chinese University of Hong Kong, Hong Kong, SAR PR China; Shenzhen Research Institute, The Chinese University of Hong Kong, Shenzhen, PR China; School of Biomedical Sciences, The Chinese University of Hong Kong, Hong Kong, PR China; Department of Medicine and Therapeutics, The Chinese University of Hong Kong, Hong Kong, PR China

**Keywords:** Gastric adenocarcinoma, MicroRNA-15a, MicroRNA-16-1, Tumor suppressor, Yes-associated protein 1

## Abstract

**Background:**

MicroRNAs (miRNAs) have been reported to play an important role in tumorigenesis. In this study, the role of miR-15a and miR-16-1 in gastric adenocarcinoma (GAC) was investigated.

**Methods:**

The expression of miR-15a and miR-16-1 in cell lines and primary tumors was examined by miRNA qRT-PCR. Proliferative assays, colony formation, cell invasion and migration, flow cytometry analysis and *in vivo* study were performed by ectopic expression of miR-15a and miR-16-1. The putative target genes of miR-15a and miR-16-1 were explored by TargetScan and further validated.

**Results:**

We found that miR-15a and miR-16-1 were down-regulated in GAC cell lines and primary tumor samples compared with normal gastric epithelium. Functional study demonstrated that ectopic expression of miR-15a and miR-16-1 suppressed cell proliferation, monolayer colony formation, invasion and migration, and xenograft formation *in vivo*. In addition, miR-15a and miR-16-1 induced G0/G1 cell cycle arrest which was further confirmed by Western blot and qRT-PCR of related cell cycle regulators. YAP1 was confirmed to be a functional target of miR-15a and miR-16-1 in GAC. YAP1 re-expression partly abrogated the inhibitory effect of miR-15a and miR-16-1 in GAC cells. In clinical samples, YAP1 protein expression shows negative correlation with miR-15a and miR-16-1 expression.

**Conclusion:**

In conclusion, targeting YAP1 by tumor suppressor miRNA miR-15a and miR-16-1 plays inhibitory effect and this might have a therapeutic potential in GAC.

**Electronic supplementary material:**

The online version of this article (doi:10.1186/s12943-015-0323-3) contains supplementary material, which is available to authorized users.

## Background

Gastric adenocarcinoma (GAC) is one of the most common malignancies worldwide, with a higher incidence in eastern Asian countries including China, Japan and South Korea. Several risk factors are involved in GAC tumorigenesis including *Helicobacter Pylori* infection, high-salt and smoked diet, smoking and chronic gastritis [1]. The genetic and epigenetic alterations of oncogenes, tumor suppressor genes and mismatch repair genes were found to play a role in gastric tumorigenesis. The tumor suppressor genes such as B cell CLL/lymphoma 6 member B (BCL6B) [2] and paired box gene 5 (PAX5) [3] were epigenetically inactivated in GAC. Some oncogenes including Stathmin 1 (STMN1) [4] and Yin Yang 1 (YY1) [5] have been reported to be over-expressed in GAC and correlated with poor survival in GAC.

MicroRNAs (miRNAs) recently have been identified as one of the crucial players in carcinogenesis through post-transcriptional regulation of their target genes [6]. miRNA is a class of small non-coding RNAs which function as regulators of gene expression through specific binding to the miRNA recognition elements (MREs) on the 3′ untranslated regions (UTRs) of target mRNAs. This results in mRNA degradation or translational repression. Emerging evidence shows that miRNAs are abnormally expressed in various cancers and the dysregulated miRNA expression is associated with tumor initiation, promotion and progression. Either overexpression of oncogenic miRNAs or downregulation of tumor suppressor miRNAs can promote tumorigenesis.

The most highly expressed miRNAs in GAC were miR-20b, miR-17, miR-18a and miR-21 whereas miR-768-3p, miR-139-5p, miR-31 and miR-195 showed decreased expressions [7]. Some miRNAs with tumor suppressor function were identified in GAC such as miR-610 targeting VASP [8], miR-7 targeting IGF1R [9], miR-625 targeting ILK [10] and let-7 targeting AKT2 [11].

We also performed miRNA expression microarray screening in 7 GAC cell lines (Additional file 1: Table S1) and miR-15a and miR-16-1 were screened out to be dramatically decreased in expression compared with normal gastric epithelium (Additional file 2: Table S2). This was further validated by miRNA qRT-PCR. Although miR-15 and miR-16-1 have been reported to play a role in the development of multi-drug resistance (MDR) of GAC cells at least by modulating apoptosis via targeting BCL2 [12], the functional role and other downstream targets of miR-15a and miR-16-1 are still not well elucidated in GAC. Therefore, we aimed to investigate the functional roles of miR-15a and miR-16-1 and to identify their novel target gene in gastric carcinogenesis.

## Results

### miR-15a and miR-16-1 are down-regulated in GAC

**Figure 1:**
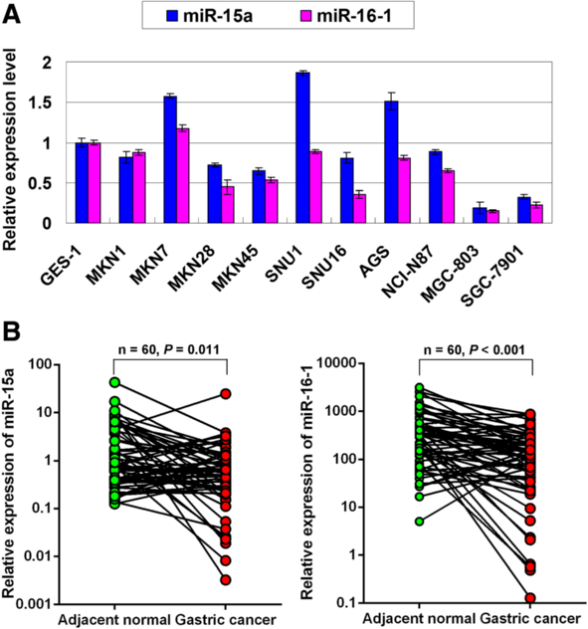
**miR-15a and miR-16-1 show decreased expression in GAC. (A)** The expression of miR-15a and miR-16-1 in 10 GAC cell lines compared with GES-1 cells. The standard deviations (SDs) were achieved by the values in triplicate wells. **(B)** Expression of miR-15a and miR-16-1 in paired primary GAC samples (n = 60; miR-15a, *P* = 0.011; miR-16-1, *P* < 0.001).

miR-15a and miR-16-1 showed decreased expression in 7 and 9 gastric cancer cell lines respectively compared with immortalized gastric epithelium cell lines GES-1 (Figure 1A). In 60 paired primary GAC samples, miR-15a and miR-16-1 showed downregulation in adenocarcinoma compared with corresponding adjacent non-tumorous mucosae (miR-15a, mean value: 1.23 Vs 3.25, *P* = 0.011; miR-16-1, mean value: 193.2 Vs 517.9, *P* < 0.001; Figure 1B).

### Ectopic expression of miR-15a and miR-16-1 exerts tumor suppressor function

**Figure 2:**
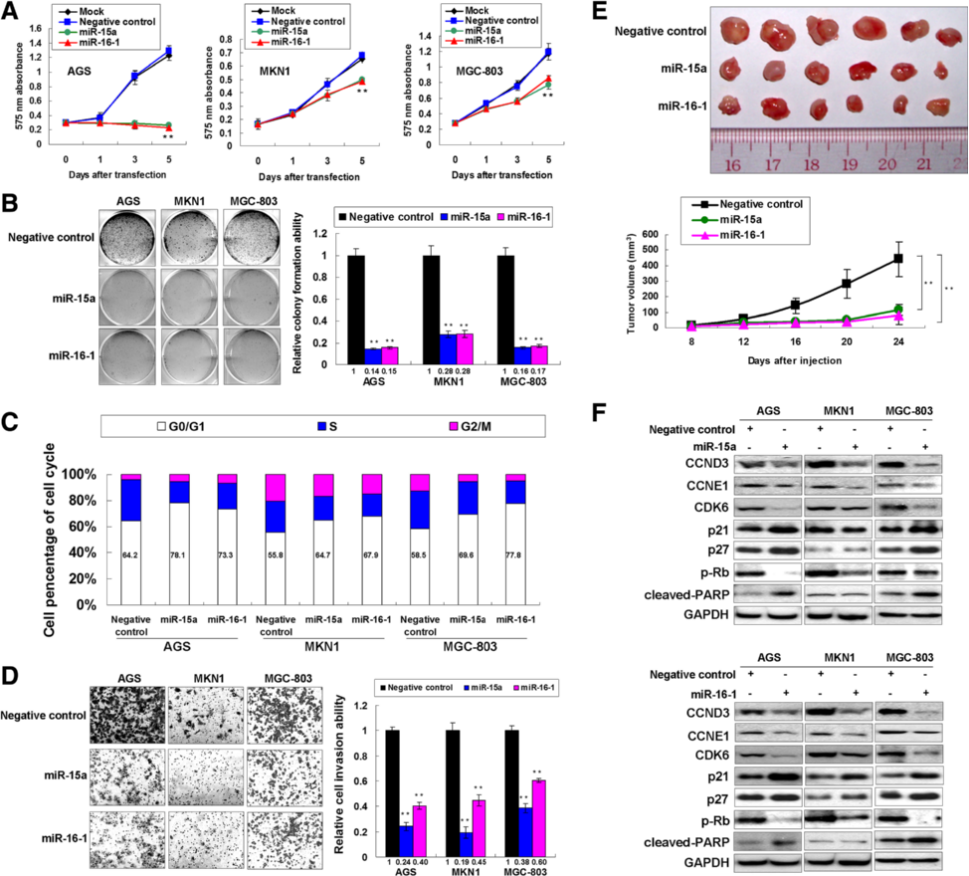
**miR-15a and miR-16-1 exert tumor suppressor function in GAC. (A)** MTT proliferation results of ectopic miR-15a and miR-16-1 expression in AGS, MKN1 and MGC-803 cells (**, *P* < 0.001). The *P*-Value was calculated by the 575 nm absorbance readings in Day 5. **(B)** Ectopic expression of miR-15a and miR-16-1 inhibited monolayer colony formation in AGS, MKN1 and MGC-803 cells (**, *P* < 0.001). The experiment was performed in triplicate wells to get SDs. **(C)** Flow cytometry analysis of miR-15a and miR-16-1 transfectants compared with scramble miRNA transfectants. Two independent experiments were performed and the representative one was shown. **(D)** miR-15a and miR-16-1 suppressed cell invasion through matrigel (**, *P* < 0.001). Representative images of cell invasion were also shown. The invaded cells in 3 random vision fields were counted and calculated to get SDs. **(E)** miR-15a-MGC-803 and miR-16-1-MGC-803 formed smaller tumors than scramble-miRNA-MGC-803 (**, *P* < 0.001). The tumor sizes in the Day 24 were calculated for the *P*-Value. **(F)** Western blot analysis of CCND3, CCNE1, CDK6, p21, p27, p-Rb and cleaved-PARP after miR-15a and miR-16-1 transfection.

Ectopic expression of miR-15a and miR-16-1 in gastric cancer cells was conducted for observation of the phenotype changes. qRT-PCR revealed that miR-15a and miR-16-1 increased to 16.4 ~ 63.8 folds in overexpression group compared with negative control group (Additional file 3: Figure S1A). Ectopic expression of miR-15a and miR-16-1 suppressed cell growth of AGS, MKN1 and MGC-803 in a 5-day MTT proliferation assay (*P* < 0.001, Figure 2A). The proliferative suppression effect of miR-15a and miR-16-1 was further validated by monolayer colony formation with a significant reduction of colony numbers in miR-15a and miR-16-1 transfectants compared with the negative groups (*P* < 0.001, Figure 2B). Ectopic expression of miR-15a and miR-16-1 resulted in G0/G1 cell cycle arrest and reduction of S phase cells (Figure 2C and Additional file 3: Figure S1B). In addition, miR-15a and miR-16-1 overexpression induced senescence which was representative by the beta-Galactosidase positive staining in a 3-day transfection assay (Additional file 3: Figure S1C). miR-15a and miR-16-1 significantly suppressed the invasion (*P* < 0.001, Figure 2D) and migration (Additional file 3: Figure S1D) abilities of GAC cells. To further explore the role of miR-15a and miR-16-1 on tumor growth *in vivo*, MGC-803 cell line which could form tumors in mouse was employed. MGC-803 cells with ectopic miR-15a and miR-16-1 expression were inoculated into the dorsal flank of nude mice. 24 days later, the xenografts with miR-15a and miR-16-1 overexpression were significantly smaller than the control group (*P* < 0.001, Figure 2E). The associated cell cycle regulators were also analyzed by Western blot. Expressions of CCND3, CCNE1, CDK6 and p-Rb were decreased but p21 and p27 were uniformly showed up-regulated in miR-15a/miR-16-1 ectopic expression cells (Figure 2F), supporting the G0/G1-phase cell cycle arrest determined by cell cycle analysis. However, the cleaved-PARP only showed activated in AGS and MGC-803 cells, suggesting that miR-15a and miR-16-1 induced late apoptosis in a cell dependant manner. The mRNA expression of some proliferation and invasion related genes such as CCND3, CCNE1, Ki-67 and MMP3 were all down-regulated in miR-15a/miR-16-1 overexpression group (Additional file 3: Figure S1E), elucidating the tumor-suppressive functions of miR-15a and miR-16-1 in gastric tumorigenesis.

### YAP1 is a direct target of miR-15a and miR-16-1 in GAC

**Figure 3:**
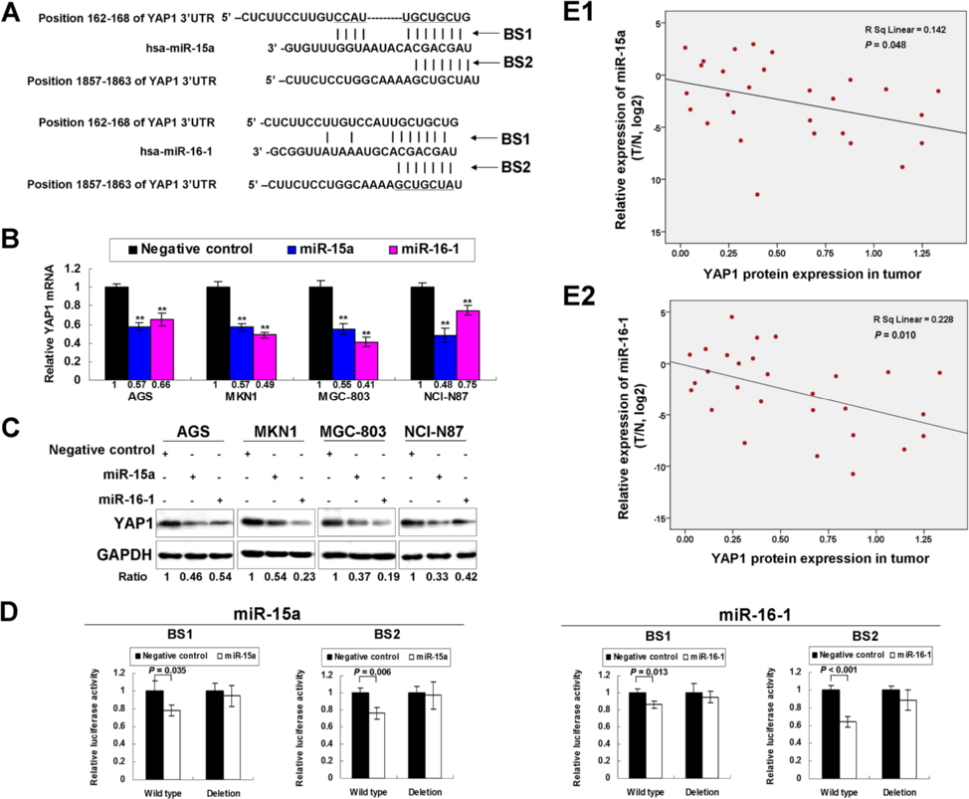
**YAP1 is a target of miR-15a and miR-16-1 in GAC. (A)** The miR-15a and miR-16-1 binding sites in YAP1 3′UTR as TargetScan predicted (www.targetscan.org). **(B)** The YAP1 mRNA expression after ectopic miR-15a and miR-16-1 expression in AGS, MKN1, MGC-803 and NCI-N87 cells (**, *P* < 0.001). **(C)** miR-15a and miR-16-1 transfection down-regulated YAP1 protein expression in GAC cells. **(D)** miR-15a and miR-16-1 suppressed the luciferase activities in the constructs containing wild-type binding sites in YAP1 3′UTR, but for the constructs containing the deletion binding sites, miR-15a and miR-16-1 had no suppressive effect on the luciferase activities. **(E1)** miR-15a showed negative correlation with YAP1 protein expression (*P* = 0.048). **(E2)** miR-16-1 expression was negatively correlated with YAP1 protein expression (*P* = 0.010).

By TargetScan (www.targetscan.org) several putative targets including YAP1 were found to have miR-15a and miR-16-1 binding site in their 3′UTR. Figure 3A demonstrated the two miR-15a and miR-16-1 binding sites in YAP1 3′UTR. The YAP1 mRNA expression showed significantly decreased in AGS, MKN1, MGC-803 and NCI-N87 cells upon ectopic miR-15a and miR-16-1 expression (*P* < 0.001, Figure 3B). YAP1 protein was also uniformly down-regulated after miR-15a/miR-16-1 transfection in all 4 GAC cell lines (Figure 3C). To investigate whether YAP1 is a direct target of miR-15a and miR-16-1, the constructs encompassing the wild type binding site in YAP1 3′UTR or deletion counterpart were co-transfected with Renilla luciferase vector into MGC-803 cells. As shown in Figure 3D, miR-15a and miR-16-1 decreased the luciferase activity in the constructs containing wild type binding site (miR-15a, binding site 1, *P* = 0.035, binding site 2, *P* = 0.006; miR-16-1, binding site 1, *P* = 0.013, binding site 2, *P* < 0.001), whereas the luciferase activities of the constructs with binding site-deletion showed no difference upon miR-15a and miR-16-1 expression compared with negative control. These results revealed that miR-15a and miR-16-1 directly bind with YAP1 3′UTR. To further validate the translational suppressive effect of miR-15a and miR-16-1 on YAP1, the anti-miR-15a and anit-miR-16-1 were transfected in two immortalized gastric epithelial cells, GES-1 and HFE-145. We found YAP1 protein showed up-regulated expression upon ectopic expression of anti-miR-15a and anti-miR-16-1 (Additional file 4: Figure S2), suggesting that YAP1 high-expression was partly due the down-regulation of miR-15a and miR-16-1. To fully recapitulate the intrinsic regulation of miR-15a and miR-16-1 on YAP1, MKN45, a cell line with negative YAP1 expression due to homozygous deletion, was used for co-transfection of YAP1 cDNA construct devoid of 3′UTR with miR-15a/miR-16-1. YAP1 protein was found no change in MKN45 after co-transfection (Additional file 5: Figure S3), suggesting that miR-15a and miR-16-1 regulate YAP1 expression through binding to its 3′UTR. As miR-15a and miR-16-1 showed decreased expression whereas YAP1 showed up-regulated expression in GAC, the expression correlations of miR-15a and miR-16-1 on YAP1 were investigated in 28 paired fresh tissues. YAP1 protein expression shows negatively correlation with miR-15a (*P* = 0.048, Figure 3E1) and miR-16-1 (*P* = 0.010, Figure 3E2). This result indicated that the downregulation of miR-15a and miR-16-1 was partly responsible for endogenous YAP1 overexpression in GAC.

### YAP1 knowdown phenocopies ectopic expression of miR-15a and miR-16-1

**Figure 4:**
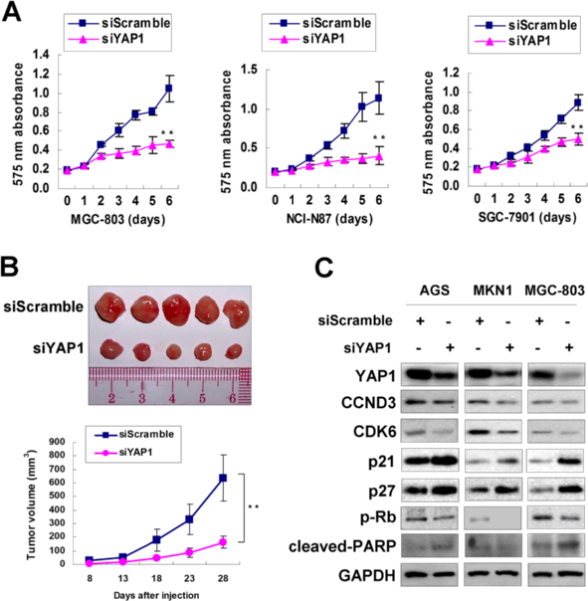
**siRNA-mediated YAP1 knockdown suppressed GAC cell proliferation** ***in vitro*** **and** ***in vivo*** **. (A)** YAP1 knockdown suppressed cell proliferation in MGC-803, NCI-N87 and SGC-7901 cells (**, *P* < 0.001). The 575 nm absorbance readings in Day 6 were calculated for the *P*-Value. **(B)** siYAP1-MGC-803 formed smaller xenografts than siScramble-MGC-803 control in nude mice (**, *P* < 0.001). The tumor sizes in the Day 28 were calculated for the *P*-Value. **(C)** YAP1 knowdown decreased the expression of CCND3, CDK6 and p-Rb, but increased the expression of p21 and p27. The cleaved-PARP showed activated expression in AGS and MGC-803 cells upon YAP1 knockdown.

As YAP1 is a direct target of miR-15a and miR-16-1 in GAC, we then checked if siRNA-mediated knockdown phenocopied the tumor-suppressive function of miR-15a and miR-16-1. Knocking down YAP1 suppressed *in vitro* cell proliferation in MGC-803, NCI-N87 and SGC-7901 cells (*P* < 0.001, Figure 4A). siYAP1-MGC-803 formed smaller xenografts than the siScramble-MGC-803 control in a 28-day *in vivo* study (*P* < 0.001, Figure 4B). Meanwhile, the related cell cycle regulators CCND3, CDK6 and p-Rb showed down-regulated expression but p21 and p27 showed up-regulated expression upon siYAP1 transfection (Figure 4C). As the same, cleaved-PARP only showed activated expression in AGS and MGC-803 cells, which was similar with the changes of miR-15a and miR-16-1 ectopic expression.

### YAP1 re-expression partly abolishes the tumor-suppressive effect of miR-15a and miR-16-1

**Figure 5:**
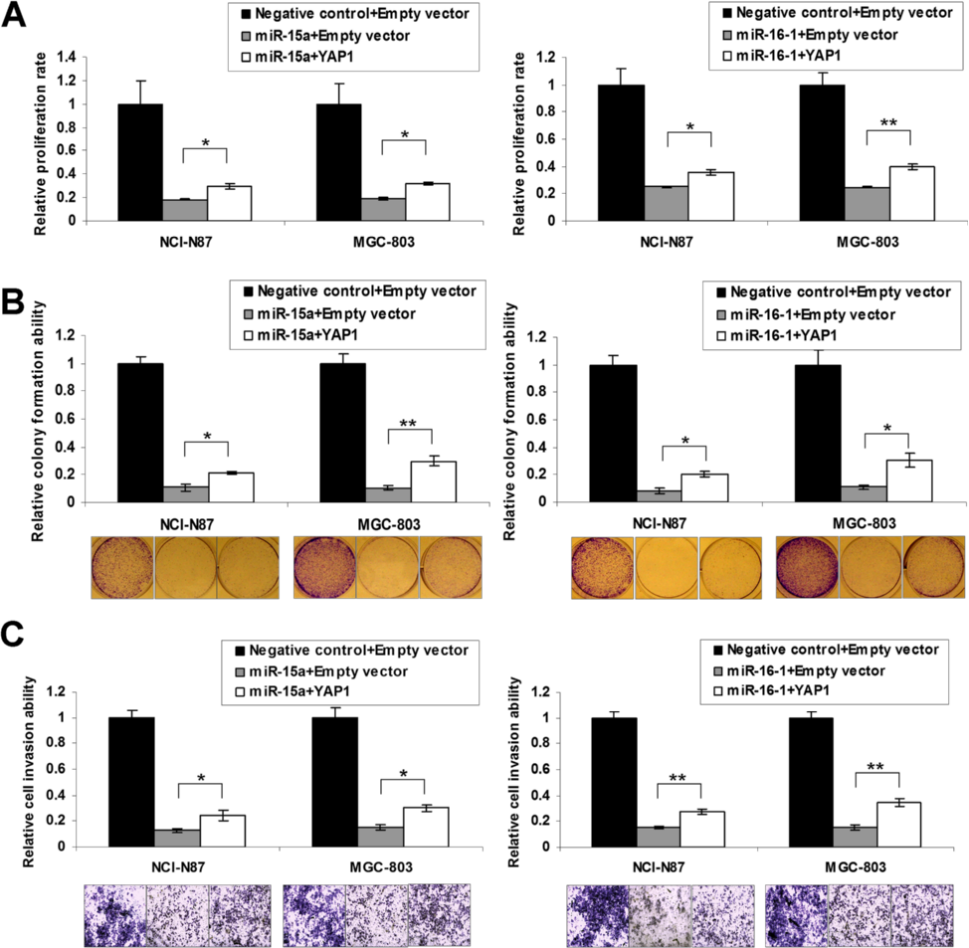
**YAP1 re-expression partly abrogated the inhibitory effect of miR-15a and miR-16-1 in NCI-N87 and MGC-803. (A)** YAP1 re-expression increased cell proliferation compared with miR-15a or miR-16-1 alone (*, *P* < 0.05; **, *P* < 0.001). The SDs were achieved by the 575 nm absorbance readings in 6 wells of each item. **(B)** Monolayer colony formation assays revealed YAP1 re-expression abolished proliferation-inhibition of miR-15a and miR-16-1 partially (*, *P* < 0.05; **, *P* < 0.001). The representative colony formation pictures were shown in the bottom. The experiments was performed in triplicate wells to get SDs. **(C)** The suppressed cell invasion abilities was partially alleviated by YAP1 re-expression in NCI-N87 and MGC-803 cells (*, *P* < 0.05; **, *P* < 0.001). The cells were counted in 3 random vision fields of each item to get SDs.

YAP1 re-expression in rescuing the suppressive phenotypes of cancer cells by ectopic expression of miR-15a and miR-16-1 was investigated. Interestingly, the growth inhibitory phenotypes were partly alleviated by YAP1 re-expression in NCI-N87 and MGC-803 cells (MTT proliferation assay, *P* < 0.05, Figure 5A; monolayer colony formation assay, *P* < 0.05, Figure 5B). The invasion-inhibitory effects of miR-15a and miR-16-1 were also partially diminished by YAP1 re-expression (*P* < 0.05, Figure 5C).

## Discussion

In this study, it was first discovered that miR-15a and miR-16-1 were consistently down-regulated across a panel of GAC cells compared with normal gastric epithelium, which suggested their potential roles in gastric tumorigenesis. In 9 gastric cancer cells, we compared miR-15a and miR-16-1 expression with the DNA copy number change of their loci (in 13q14.2, from array-CGH result of gastric cancer cell lines) and found their expression shows no positive correlation the DNA copy number change (Additional file 6: Figure S4A). We also treated the gastric cancer cells with 5-Aza and TSA to investigate if epigenetic modification is responsible for the downregulation of miR-15a/16-1 in GAC, however, miR-15a and miR-16-1 showed no significant expression change (>2 fold change) upon treatment in NCI-N87 and MGC-803 cells (Additional file 6: Figure S4B). So the mechanisms of miR-15a and miR-16-1 downregulation in GAC need further investigation. Ectopic expression of miR-15a and miR-16-1 exerted tumor suppressor function by inhibiting GAC cell proliferation *in vitro* and *in vivo*, inhibiting cell invasion and migration, induced G0/G1 phase cell cycle arrest even caused senescence. These functional studies suggested that miR-15a and miR-16-1 played a crucial role in cellular homeostasis, when dysregulated, might contribute to the development of GAC.

miR-15a and miR-16-1, encoded by the miR-15/16-1 cluster, were identified as tumor suppressors . Expression of these miRNAs inhibits cell proliferation, promotes apoptosis of cancer cells and suppresses tumorigenicity both *in vitro* and *in vivo* [13]. Downregulation of these two miRNAs has been well elucidated in chronic lymphocytic lymphoma (CLL) [14]. Deletions in chromosome 13 together with histone deacetylases overexpression account for the loss of expression of miR-15a and miR-16-1 in CLL [15]. The miR-15a and miR-16-1 expression were found to be inversely correlated with BCL2 [16] and WT1 oncogene expression in CLL [17]. miR-15a and miR-16-1 were down-regulated in fibroblasts surrounding the prostate tumors and their downregulation resulted in the development and progression of prostate cancer [18]. Up-regulated BCL2, CCND1 and WNT3A which promote tumorigenic features, are the main targets of miR-15a and miR-16-1 in prostate cancer [19]. In non small cell lung carcinoma (NSCLC), miR-15a and miR-16-1 cluster regions were frequently deleted or their expressions were down-regulated. Expression of miR-15a and miR-16-1 have been found to inversely correlate with the expression of cell cycle regulators CCND1 and CCNE1 [20]. In addition, these miRNAs were shown to induce Rb-dependent cell cycle arrest in NSCLC [21]. Downregulation of miR-15a has also been reported to enhance proliferation on pancreatic cancer cells [22,23]. Apart from this, miR-15a and miR-16-1 were able to induce apoptosis of rat pancreatic stellate cells by inhibiting BCL2 expression [24]. In ovarian cancer, the miR-15a and miR-16-1 levels were found to be inversely correlated to the protein expression levels of Bmi-1 which its 3′UTR is the direct target of these miRNAs [25]. BCL2, an anti-apoptotic protein and important target of miR-15/16, was also found to be negatively regulated by miR-15a and miR-16-1 in GAC (Additional file 3: Figure S1F). Our functional study enriched the tumor suppressive role of miR-15a and miR-16-1 in various cancer types.

From the target gene screening, YAP1, which was also negatively regulated by miR-375 [26], was first identified as a novel downstream target of miR-15a and miR-16-1 in gastric cancer. Our previous study showed that YAP1 exerted oncogenic function in GAC development by constitutively activating RAF/MEK/ERK pathway. YAP1 overexpression promoted anchorage independent colony formation, induced a more invasive phenotype and accelerated cell growth both *in vitro* and *in vivo* [27]. Anti-YAP1 siRNA suppressed cell proliferation, decreased cell invasion and colony formation ability and induce G0/G1 cell cycle arrest, which resembled the growth-inhibitory phenotypes of miR-15a and miR-16-1 in GAC cells. We further confirmed YAP1 re-expression partly abrogated the inhibitory effect of miR-15a and miR-16-1. In primary tumors, YAP1 protein expression shows negative correlation with miR-15a and miR-16-1 expression. Collectively, these data supported that YAP1 was a main target of miR-15a and miR-16-1 in gastric tumorigenesis.

In summary, our results revealed that miR-15a and miR-16-1, which functions as tumor suppressors, were down-regulated in GAC. Ectopic expression of miR-15a and miR-16-1 suppressed GAC cell proliferation, at least in partial by targeting YAP1 oncoprotein. These findings suggested that the frequently down-regulated miR-15a and miR-16-1 in GAC contributed to GAC progression and therefore miR-15a and miR-16-1 might have a therapeutic potential for GAC treatment. At the mean time, our findings provided additional details for the post-transcriptional regulation of YAP1 besides the classic Hippo signaling pathway.

## Materials and methods

### Cell lines and primary gastric cancer tissues

Human GAC cell lines (MKN1, MKN7, MKN28, MKN45, SNU1, SNU16, AGS, NCI-N87, MGC-803, SGC-7901) (Additional file 7: Table S3) and two immortalized gastric epithelial cells (GES-1 and HFE-145) were employed as in our previous report [4]. Cells were cultured at 37°C in a humidified air atmosphere containing 5% carbon dioxide in RPMI 1640 medium (GIBCO, Grand Island, NY) supplemented with 10% fetal bovine serum (GIBCO) The primary paired samples (tumor samples and adjacent non-tumorous samples) from GAC patients were randomly selected from Prince of Wales Hospital. Ethical approval was obtained from the Joint Chinese University of Hong Kong-New Territories East Cluster Clinical Research Ethics Committee (CREC Ref. No.2009.521).

### RNA extraction and quantitative real-time polymerase chain reaction (qRT-PCR)

A total of 32 paired formalin-fixed and paraffin-embedded and 28 paired frozen GAC samples were included in this study. Tissues from formalin-fixed and paraffin-embedded sections were isolated by micro-dissection under microscope and total RNA was extracted using RecoverAll Total Nucleic Acid Isolation Kit (Ambion, Austin, TX). Total RNA from fresh tissue samples and cultured cells was extracted using TRIzol reagent (Invitrogen, Carlsbad, CA), High-Capacity cDNA Reverse Transcription Kit (Applied Biosystems, Carlsbad, CA) was used for cDNA synthesis. qRT-PCR was used to quantify differences in mRNA expression and primers were listed in Table S4 (Additional file 8). The relative expression level was normalized by RPL29 in gastric tissues and B2M in gastric cancer cell lines [28]. PCR was performed using SYBR Green PCR reagents (Applied Biosystems) according to the manufacturer’s instructions. The reactions were incubated in a 96-well plate at 95°C for 10 min, followed by 40 cycles of 95°C for 15 seconds and 60°C for 1 minute.

For miRNA expression detection, Taqman miRNA assays were used to quantify the expression levels of mature miR-15a and miR-16-1 (000389 and 000391, Applied Biosystems). The relative expression level of microRNAs was normalized by RNU6B (001093, Applied Biosystems). The reactions were performed in 7500 Fast Real-Time System (Applied Biosystems) and the reaction mix was incubated at 95°C for 30 seconds, followed by 40 cycles of 95°C for 8 seconds and 60°C for 30 seconds [11].

### Protein extraction and Western blot analysis

Protein was extracted from GAC cell lines and paired primary tissues using RIPA lysis buffer with proteinase inhibitor. Protein concentration was measured by the method of Bradford (Bio-Rad, Hercules, CA) and 20 μg of protein mixed with 2 × SDS loading buffer was loaded per lane, separated by 12% SDS-polyacrylamide gel electrophoresis. Horseradish peroxidase (HRP) substrate solution was used for signal detection (Millipore, Billerica, MA). YAP1 protein was detected with a monoclonal anti-YAP1 antibody (1:10000 dilution, ab52771, Abcam, Cambridge, MA). Other primary antibodies were obtained from Cell Signaling Technology (Danvers, MA), CCND3 (1:2000, #2936), CCNE1 (1:1000, #4129), CDK6 (1:2000, #3136), p21 (1:1000, #2946), p27 (1:1000, #2552), p-Rb(Ser807/811) (1:1000, #9308), cleaved PARP(Asp214) (1:1000, #9541), BCL2(1:1000, #2870). The secondary antibodies were anti-Mouse IgG-HRP (1:30000 dilution, 00049039, Dako, Glostrup, Denmark) and anti-Rabbit IgG-HRP (1:10000, 00028856, Dako). The Western blot bands were quantified by ImageJ.

### miRNA/siRNA transfection and functional study

Transfection of miR-15a and miR-16-1 precursors (PM10235 and PM10339, Applied Biosystems) and scramble control (AM17110) was performed using Lipofectamine 2000 Transfection Reagent (Invitrogen). All transfection were performed in a 30 nM concentration for 36 hours followed with functional study and RNA/protein analysis. MTT cell proliferation was assessed by CellTiter 96 Non-Radioactive Cell Proliferation Assays (Promega, Madison, WI) according to manufacturer’s instruction. Four groups of cells were evaluated: miR-15a, miR-15a precursor transfection; miR-16-1, miR-16-1 precursor transfection; Mock control, only Lipofectamine; Negative control, scramble miRNA transfection. For colony formation assays in monolayer cultures, cells transfected with miRNA were cultured for 14 days, fixed with 70% ethanol for 15 minutes and stained with 2% crystal violet. Colonies with more than 50 cells per colony were counted. The experiments were repeated in triplicate to get standard deviations. The cell migration assays were performed by Transwell Polycarbonate Membrane Inserts (Corning, NY). The cell invasion assay using BD Biocoat Matrigel Invasion Chambers (BD Biosciences, Franklin Lakes, NJ) has been described previously [27]. The functional study of siYAP1 (SI02662954, Qiagen, Valencia, CA) was also performed as above except that a concentration of 25 nM siRNA was used.

In the senescence experiments, AGS, MKN1, MGC-803 cells were transfected with miR-15a, miR-16-1 or negative control for 3 days in 20 nM concentration. Then the cells were stained by senescence beta-Galactosidase (Kit, #9860, Cell Signaling) for 8 hours and the positive cell population showed pale green. The positive cell was counted under microscope and the standard deviation was achieved by calculated the ratio of positive cells per 100 cancer cells in three random vision fields and normalized by the negative control group.

For the rescue experiments, miR-15a and miR-16-1 precursors and the negative control were separately transfected into NCI-N87 and MGC-803 cells. And 24 hours after precursor transfection, YAP1 expression plasmid (pcDNA3.1-YAP1) or empty vector (pcDNA3.1, Life Technologies) were subsequently transfected with FuGENE HD Transfection Reagent (Roche, Nutley, NJ). After another 24 hours, cells were harvested for functional study (MTT proliferation assays, monolayer colony formation assays and cell invasion assays).

### Cell cycle analysis

Cell cycle analysis was performed using flow cytometry as described previously [5].

### *In vivo* tumorigenicity study

MGC-803 cells (1 × 10^7^ cells suspended in 0.1 ml PBS) transiently transfected with scramble control or miR-15a/miR-16-1 were injected subcutaneously into the dorsal flank of six 4-week-old Balb/c nude mice. The tumor diameter was measured and documented to get tumor volume every 4 days form day 8. 24 days later, the mice were sacrificed and the xenografts were taken out. Tumor volume (mm^3^) was estimated by measuring the longest and shortest diameter of the tumor and calculating as follows: volume = (shortest diameter)^2^ × (longest diameter) × 0.5.

For siYAP1 *in vivo* study, the procedures were the same as above. MGC-803 cells transfected with siYAP1 were implanted on the right dorsal flank and cells transfected with siScramble were implanted on the left dorsal flank of nude mice. The tumor diameter was documented every 5 days from day 8 to day 28.

All animal handling and experimental procedures were approved by Department of Health, Hong Kong (Reference No: 14–267 in DH/HA&P/8/2/1 Pt.38).

### Luciferase assays

The two putative miR-15a and miR-16-1 MREs in YAP1 3′UTR were subcloned into pMIR-REPORT vector (Ambion). Two deletion constructs were generated by deletion of the complementary entire seed sequence of miR-15a and miR-16-1. The sense and anti-sense of oligonucleotides (Additional file 9: Table S5) that encompassed the miR-15a and miR-16-1 binding sites were annealed and inserted into the vector [29]. The firefly luciferase constructs were co-transfected with the Renilla luciferase vector (Promega) control into MGC-803 cells with FuGENE HD Transfection Reagent. Dual Luciferase Reporter Assay System (Promega) was employed to measure the luciferase activity for normalization 24 hours after transfection.

### Statistical analysis

The Student T test was used to compare the difference in biological behavior between miR-15a and miR-16-1 transfected cells and scramble miRNA control transfected cells. Expression of miR-15a/miR-16-1 in GAC cell lines, primary cancerous tissues and the corresponding paired noncancerous tissues were compared by Mann–Whitney U test and paired T test. The miR-15a/16-1 expression in gastric cancer cell lines was compared with its copy number change by non-parametric Spearman’s rho rank test. All statistical analysis was performed by SPSS software (Version 16.0; SPSS Inc). A two-tailed *P*-value of less than 0.05 was considered statistically significant.

## Additional files

Additional file 1: Table S1. The raw data of miRNA expression microarray in 7 gastric adenocarcinoma cell lines. Additional file 2: Table S2. The expression of miR-15a and miR-16-1 in gastric cancer cells (log2 ratio). The data was from microRNA expression microarray and normal gastric epithelium control is from Ambion (AM7996, Grand Island, NY). Additional file 3: Figure S1. Additional functional study of ectopic expression of miR-15a and miR-16-1 in gastric cancer cells (*, *P* < 0.05; **, *P* < 0.001). (A) qRT-PCR of miR-15a and miR-16-1 in AGS, MKN1 and MGC-803 after ectopic expression. (B) Representative cell cycle distribution images of FACS flow cytometry analysis in GAC cell lines upon overexpression of miR-15a and miR-16-1. (C) Overexpression of miR-15a and miR-16-1 induced senescence in a 3-day transfection assay. (D) The cell migration ability was significantly inhibited by ectopic expression of miR-15a and miR-16-1. (E) qRT-PCR analysis of CCND3, CCNE1, Ki67 and MMP3 upon ectopic expression of miR-15a and miR-16-1. (F) Overexpression of miR-15a and miR-16-1 also decreased the expression of Bcl-2 protein (a putative target of miR-15a/16-1) in GAC cell lines, but the autophagy related proteins, p62 and LC3, show no change. Additional file 4: Figure S2. anti-miR-15a and anti-miR-16-1 increased YAP1 protein expression in GES-1 and HFE-145 cells. Additional file 5: Figure S3. miR-15a and miR-16-1 had no down-regulation effect on the YAP1 expression in MKN45-YAP1 cells (devoid YAP1 3′UTR). Additional file 6: Figure S4. The genetic and epigenetic investigation of miR-15a and miR-16-1 in GAC. (A) The correlation analysis of miR-15a and miR-16-1 expression with its copy number change (from array-CGH data, n = 9; miR-15a, *P* = 0.802; miR-16-1, *P* = 0.894). (B) The expression of miR-15a and miR-16-1 after treatment with 5-Aza and TSA in NCI-N87 and MGC-803 cells. Additional file 7: Table S3. The biological information of 10 gastric cancer cell lines. Additional file 8: Table S4. Primers used in this study. Additional file 9: Table S5. Oligonucleotides used in the miR-15a/miR-16-1 targeting YAP1 3′UTR luciferase activity experiments. Wild type, miR-15a/miR-16-1 binding site in YAP1 3′UTR; Deletion, the complementary sequence of miR-15a/miR-16-1 seed region was deleted.

## Abbreviations

GAC: gastric adenocarcinoma
miRNA: microRNA
miR-15a: microRNA-15a
miR-16-1: microRNA-16-1
MREs: miRNA recognition elements
PBS: phosphate buffered saline
qRT-PCR: quantitative real-time polymerase chain reaction
SDS: sodium dodecyl sulfate
UTR: untranslated region
YAP1: Yes-associated protein 1
