# Supplementary material for: Targeting of YAP1 by microRNA-15a and microRNA-16-1 exerts tumor suppressor function in gastric adenocarcinoma
Source: Mol Cancer. 2015 Feb 22;14:52. doi: 10.1186/s12943-015-0323-3 (PMC4342823; doi:10.1186/s12943-015-0323-3)
Supplement: Additional file 2: Table S2. — The expression of miR-15a and miR-16-1 in gastric cancer cells (log2 ratio). The data was from microRNA expression microarray and normal gastric epithelium control is from Ambion (AM7996, Grand Island, NY). [file 12943_2015_323_MOESM2_ESM.doc]

**Table S2. The expression of miR-15a and miR-16-1 in gastric cancer cells (log2 ratio).** The data was from microRNA expression microarray and normal gastric epithelium control is from Ambion (AM7996, Grand Island, NY).

|  | AGS | MKN1 | MKN28 | MKN45 | SNU16 | MKN7 | SNU1 | Normal control |
| --- | --- | --- | --- | --- | --- | --- | --- | --- |
| miR-15a | -119.09 | -116.4 | -79.41 | -65.4 | 58 | -121.3 | -21.9 | 0 |
| miR-16-1 | -746.8 | -698.8 | -544.6 | -503.2 | -171.3 | -705.3 | -283.5 | 0 |
