# Supplementary figures and images for: Targeting of YAP1 by microRNA-15a and microRNA-16-1 exerts tumor suppressor function in gastric adenocarcinoma
Source: Mol Cancer. 2015 Feb 22;14:52. doi: 10.1186/s12943-015-0323-3 (PMC4342823; doi:10.1186/s12943-015-0323-3)

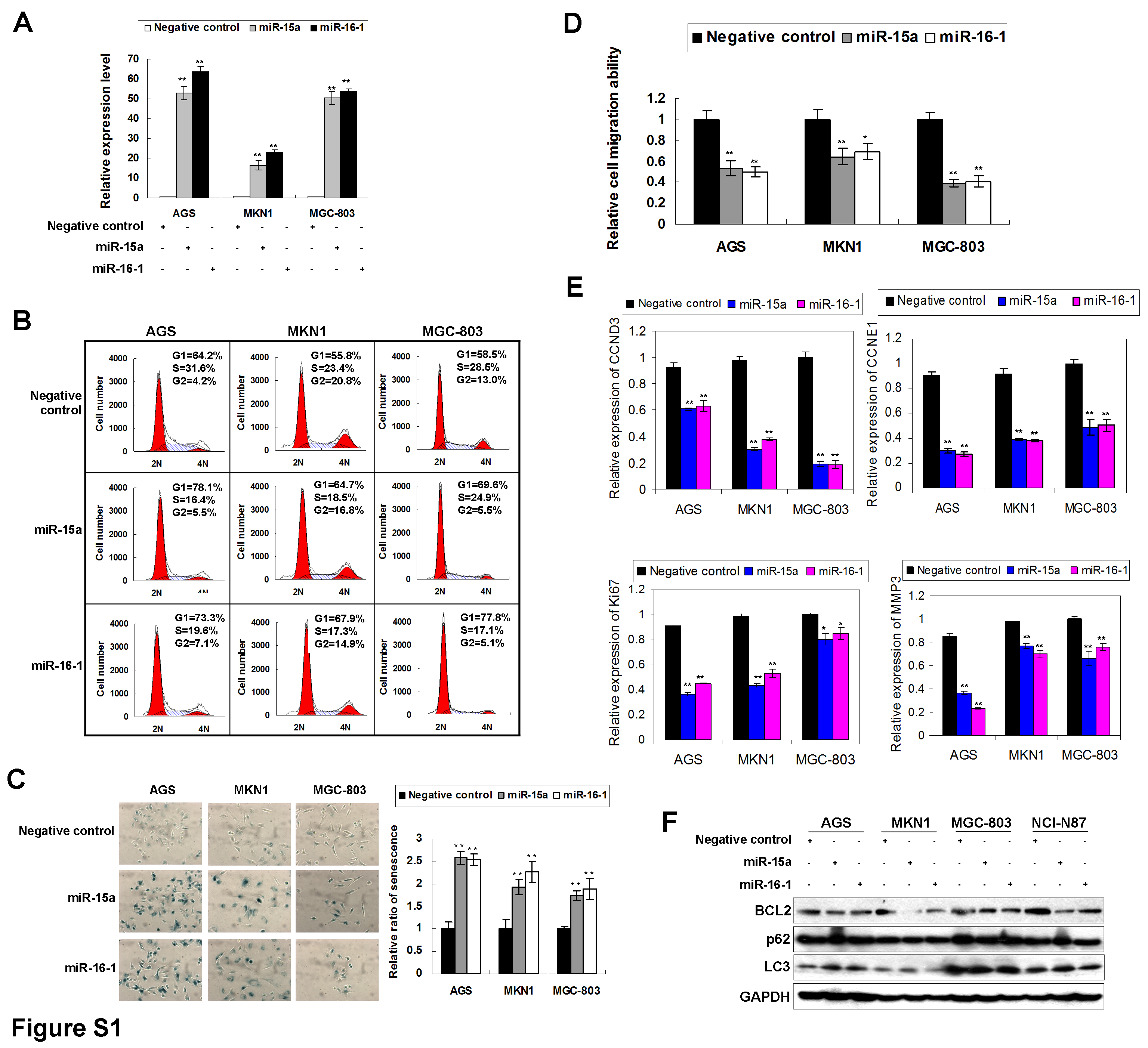

Supplement: Additional file 3: Figure S1. — Additional functional study of ectopic expression of miR-15a and miR-16-1 in gastric cancer cells (*, P < 0.05; **, P < 0.001). (A) qRT-PCR of miR-15a and miR-16-1 in AGS, MKN1 and MGC-803 after ectopic expression. (B) Representative cell cycle distribution images of FACS flow cytometry analysis in GAC cell lines upon overexpression of miR-15a and miR-16-1. (C) Overexpression of miR-15a and miR-16-1 induced senescence in a 3-day transfection assay. (D) The cell migration ability was significantly inhibited by ectopic expression of miR-15a and miR-16-1. (E) qRT-PCR analysis of CCND3, CCNE1, Ki67 and MMP3 upon ectopic expression of miR-15a and miR-16-1. (F) Overexpression of miR-15a and miR-16-1 also decreased the expression of Bcl-2 protein (a putative target of miR-15a/16-1) in GAC cell lines, but the autophagy related proteins, p62 and LC3, show no change. [file 12943_2015_323_MOESM3_ESM.tif]

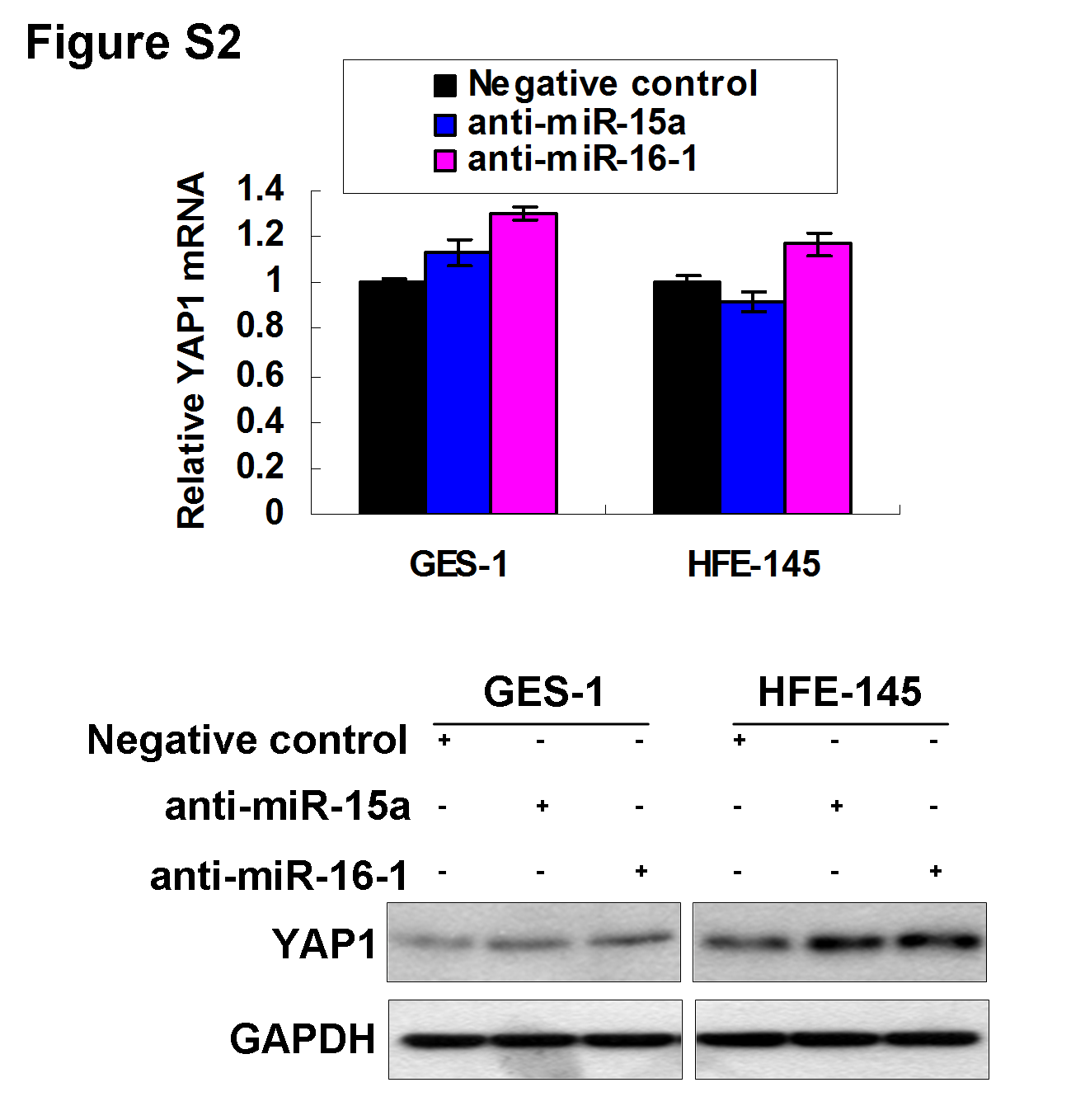

Supplement: Additional file 4: Figure S2. — anti-miR-15a and anti-miR-16-1 increased YAP1 protein expression in GES-1 and HFE-145 cells. [file 12943_2015_323_MOESM4_ESM.tif]

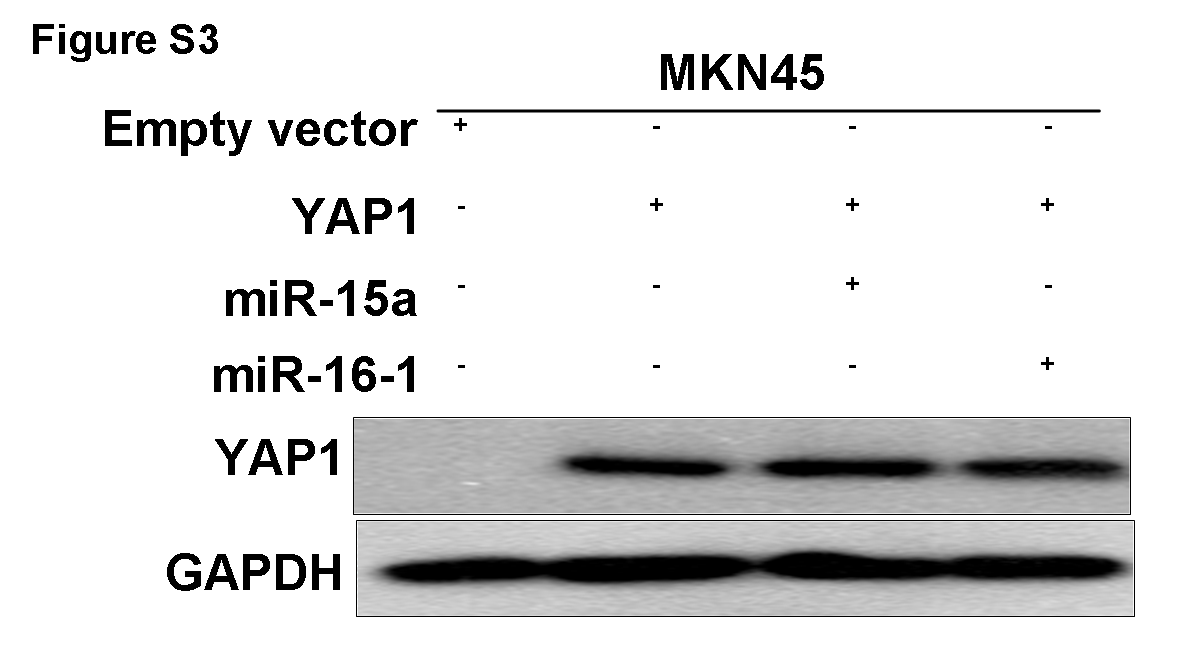

Supplement: Additional file 5: Figure S3. — miR-15a and miR-16-1 had no down-regulation effect on the YAP1 expression in MKN45-YAP1 cells (devoid YAP1 3′UTR). [file 12943_2015_323_MOESM5_ESM.tif]

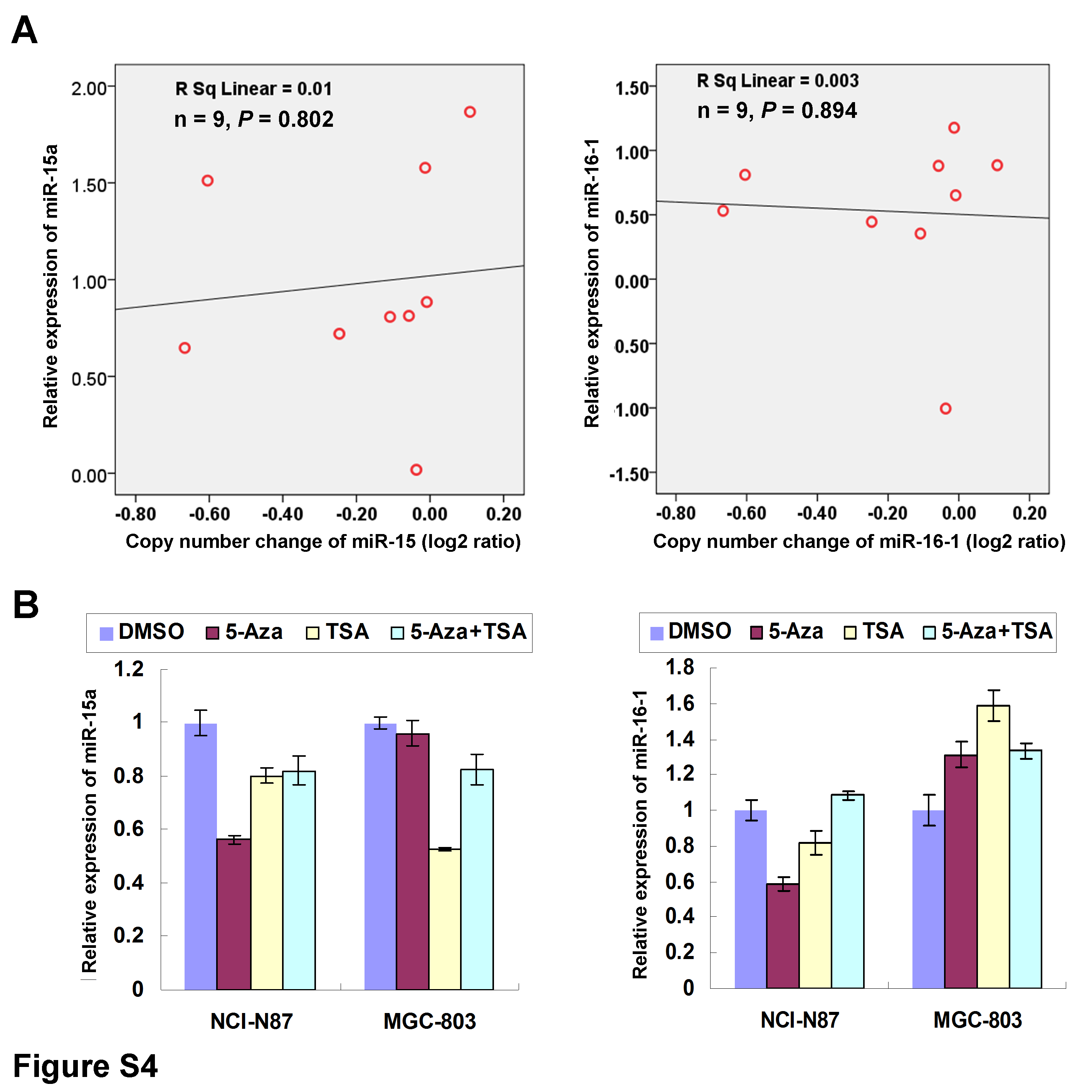

Supplement: Additional file 6: Figure S4. — The genetic and epigenetic investigation of miR-15a and miR-16-1 in GAC. (A) The correlation analysis of miR-15a and miR-16-1 expression with its copy number change (from array-CGH data, n = 9; miR-15a, P = 0.802; miR-16-1, P = 0.894). (B) The expression of miR-15a and miR-16-1 after treatment with 5-Aza and TSA in NCI-N87 and MGC-803 cells. [file 12943_2015_323_MOESM6_ESM.tif]
