# Supplementary material for: Targeting of YAP1 by microRNA-15a and microRNA-16-1 exerts tumor suppressor function in gastric adenocarcinoma
Source: Mol Cancer. 2015 Feb 22;14:52. doi: 10.1186/s12943-015-0323-3 (PMC4342823; doi:10.1186/s12943-015-0323-3)
Supplement: Additional file 7: Table S3. — The biological information of 10 gastric cancer cell lines. [file 12943_2015_323_MOESM7_ESM.doc]

**Table S3. The biological information of 10 gastric cancer cell lines.**

|  | **Cell Line** | **Gender** | **Histology** | **TP53 status** | **Xenograft** | **Growth** |
| --- | --- | --- | --- | --- | --- | --- |
| 1 | MKN1 | Male | Well differentiated, adenosquamous | Mutant | No | Adherent |
| 2 | MKN7 | Male | Well-differentiated tubular adenocarcinoma | Mutant | No | Adherent |
| 3 | MKN28 | Female | Well-differentiated tubular adenocarcinoma | Mutant | No | Adherent |
| 4 | MKN45 | Female | Poorly differentiated adenocarcinoma | Wild type | Yes | Adherent |
| 5 | SNU1 | Male | Poorly differentiated adenocarcinoma | Wild type | No | Suspension |
| 6 | SNU16 | Female | Poorly differentiated adenocarcinoma | Mutant | No | Suspension |
| 7 | AGS | Female | Moderate / poorly differentiated | Wild type | No | Adherent |
| 8 | NCI-N87 | Male | Well-differentiated adenocarcinoma | Mutant | No | Adherent |
| 9 | MGC-803 | Male | Poorly differentiated adenocarcinoma | Mutant | Yes | Adherent |
| 10 | SGC-7901 | Female | Poorly differentiated adenocarcinoma | Mutant | No | Adherent |
