# Supplementary material for: Targeting of YAP1 by microRNA-15a and microRNA-16-1 exerts tumor suppressor function in gastric adenocarcinoma
Source: Mol Cancer. 2015 Feb 22;14:52. doi: 10.1186/s12943-015-0323-3 (PMC4342823; doi:10.1186/s12943-015-0323-3)
Supplement: Additional file 8: Table S4. — Primers used in this study. [file 12943_2015_323_MOESM8_ESM.doc]

**Table S4.** Primers used in this study.

| Primers | Sense (5’-3’) | Anti-sense (5’-3’) |
| --- | --- | --- |
| YAP1 | CAGCAACTGCAGATGGAGAA | ACATCCCGGGAGAAGACACT |
| RPL29 | GGACCCCAAGTTCCTGAGG | GCATTGTTGGCCTGCATCTT |
| B2M | ACTCTCTCTTTCTGGCCTGG | ATGTCGGATGGATGAAACCC |
| CCND3 | TGACCATCGAAAAACTGTGC | GAATGAAGGCCAGGAAATCA |
| CCNE1 | TACCCAAACTCAACGTGCAA | CCTCTCTATTTGCCCAGCTC |
| Ki67 | GCTCGACCCTACAGAGTGCT | GGGGTCTTGAACATTTCAGC |
| MMP3 | GGCCAGGGATTAATGGAGAT | CAATTTCATGAGCAGCAACG |
