# Supplementary material for: Targeting of YAP1 by microRNA-15a and microRNA-16-1 exerts tumor suppressor function in gastric adenocarcinoma
Source: Mol Cancer. 2015 Feb 22;14:52. doi: 10.1186/s12943-015-0323-3 (PMC4342823; doi:10.1186/s12943-015-0323-3)
Supplement: Additional file 9: Table S5. — Oligonucleotides used in the miR-15a/miR-16-1 targeting YAP1 3′UTR luciferase activity experiments. Wild type, miR-15a/miR-16-1 binding site in YAP1 3′UTR; Deletion, the complementary sequence of miR-15a/miR-16-1 seed region was deleted. [file 12943_2015_323_MOESM9_ESM.doc]

**Table S5. Oligonucleotides used in the miR-15a/miR-16-1 targeting YAP1 3’UTR luciferase activity experiments.** Wild type, miR-15a/miR-16-1 binding site in YAP1 3’UTR; Deletion, the complementary sequence of miR-15a/miR-16-1 seed region was deleted.

| Oligonucleotide | Sense (5’-3’) | Anti-sense (5’-3’) |
| --- | --- | --- |
| Binding site 1  Wild type | CTAGTCTCTTCCTTGTCCATTGCTGCTG A | AGCTTCAGCAGCAATGGACAAGGAAGAGA |
| Binding site 1  Deletion | CTAGTCTCTTCCTTGTCCATG A | AGCTTCATGGACAAGGAAGAGA |
| Binding site 2  Wild type | CTAGTCTTCTCCTGGCAAAAGCTGCTAT A | AGCTTATAGCAGCTTTTGCCAGGAGAAGA |
| Binding site 2  Deletion | CTAGTCTTCTCCTGGCAAAAT A | AGCTTATTTTGCCAGGAGAAGA |
